# Design of Laser Activated Antimicrobial Porous Tricalcium Phosphate-Hydroxyapatite Scaffolds for Orthopedic Applications

**DOI:** 10.3390/jfb15020036

**Published:** 2024-01-30

**Authors:** Emil Filipov, Ridvan Yildiz, Anna Dikovska, Lamborghini Sotelo, Tharun Soma, Georgi Avdeev, Penka Terziyska, Silke Christiansen, Anne Leriche, Maria Helena Fernandes, Albena Daskalova

**Affiliations:** 1 Institute of Electronics, Bulgarian Academy of Sciences, 72 Tsarigradsko Chaussee Blvd., 1784 Sofia, Bulgaria; dikovska@ie.bas.bg; 2 CERAMATHS—Laboratoire de Matériaux Céramiques et de Mathématiques, Département Matériaux et Procédés, University Polytechnique Hauts-de-France, F-59313 Valenciennes, France; ridvan.yildiz@uphf.fr (R.Y.); aleriche@uphf.fr (A.L.); 3 Institute for Nanotechnology and Correlative Microscopy eV INAM, Äußere Nürnberger Str. 62, 91301 Forchheim, Germany; lamborghini.sotelo@inam-forchheim.de (L.S.); tharun.soma@inam-forchheim.de (T.S.); silke.christiansen@ikts.fraunhofer.de (S.C.); 4 Institute for Optics, Information and Photonics, Department of Physics, Friedrich-Alexander University Erlangen-Nürnberg, Staudtstraße 7, 91058 Erlangen, Germany; 5 Institute of Physical Chemistry, Bulgarian Academy of Sciences, Acad. Georgi Bonchev Str. Bld. 11, 1113 Sofia, Bulgaria; g_avdeev@ipc.bas.bg; 6 G. Nadjakov Institute of Solid State Physics, Bulgarian Academy of Sciences, Tsarigradsko Chausse 72 Blvd, 1784 Sofia, Bulgaria; penka@issp.bas.bg; 7 Fraunhofer Institute for Ceramic Technologies and Systems IKTS, Äußere Nürnberger Str. 62, 91301 Forchheim, Germany; 8 Institut für Experimentalphysik, Fachbereich Physik, Frei Universität Berlin, Arnimalle 14, 14195 Berlin, Germany; 9 Faculdade de Medicina Dentária, Universidade do Porto, Rua Dr. Manuel Pereira da Silva, 4200-393 Porto, Portugal; mhfernandes@fmd.up.pt; 10 LAQV/REQUIMTE, University of Porto, 4160-007 Porto, Portugal

**Keywords:** antimicrobial, femtosecond laser processing, bone tissue engineering, pulsed laser deposition

## Abstract

The field of bone tissue engineering is steadily being improved by novel experimental approaches. Nevertheless, microbial adhesion after scaffold implantation remains a limitation that could lead to the impairment of the regeneration process, or scaffold rejection. The present study introduces a methodology that employs laser-based strategies for the development of antimicrobial interfaces on tricalcium phosphate–hydroxyapatite (TCP-HA) scaffolds. The outer surfaces of the ceramic scaffolds with inner porosity were structured using a femtosecond laser (λ = 800 nm; τ = 70 fs) for developing micropatterns and altering local surface roughness. The pulsed laser deposition of ZnO was used for the subsequent functionalization of both laser-structured and unmodified surfaces. The impact of the fs irradiation was investigated by Raman spectroscopy and X-ray diffraction. The effects of the ZnO-layered ceramic surfaces on initial bacterial adherence were assessed by culturing *Staphylococcus aureus* on both functionalized and non-functionalized scaffolds. Bacterial metabolic activity and morphology were monitored via the Resazurin assay and microscopic approaches. The presence of ZnO evidently decreased the metabolic activity of bacteria and led to impaired cell morphology. The results from this study have led to the conclusion that the combination of fs laser-structured surface topography and ZnO could yield a potential antimicrobial interface for implants in bone tissue engineering.

## 1. Introduction

The healing of bone defects resulting from injury, infection or the removal of malignant formations remains a major challenge in orthopedic surgery. Current standards for treatment include the use of metallic implants (e.g., titanium and its alloys) or bone autografts [1]. The major disadvantages of the autografts are the limited bone tissue availability, morbidity, and an increased probability of developing a post-operational infection. Metallic implants remain a gold standard for traumas at load-bearing sites. Their properties include high tensile and compressive strength, sufficient hardness and fracture toughness [2]. Titanium and titanium alloys have been the most common choices for the replacement of joints or for the fixation of a bone plate. These materials possess good biocompatibility and elastic modulus, and a higher strength-to-weight ratio [2]. Furthermore, the formation of a stable oxide layer on the surface contributes to an increased corrosion resistance [3]. However, in cases of a weaker oxide layer, a sudden release of metallic ions could occur, which would interact with the surrounding host tissue. The local build-up of ions could result in toxicity and inflammation that could in turn compromise the implant [4]. Despite their great advantages, metallic implants’ failure could also be related to a chronic bacterial infection as a result of a gradual bacterial adhesion onto the surface of the implants, and the formation of a biofilm. Such complications have been reported to occur in between 1% and 2% of primary joint replacement surgeries and at about 3% to 5% during revision surgeries [5]. The most common pathogens causing implant infections are *S. aureus* and *S. epidermidis*, accounting for approximately 70% of cases [5]. Bacterial biofilms can be defined as the assembly of microbial cells on a surface embedded in an extracellular polymeric substance (EPS) [6]. The adherence and the subsequent embedding of pathogens on the implant surface could occur both during a surgery or during the post-operative period, as planktonic opportunistic bacteria within the host would compete with the host cells. There are various factors that could contribute to bacterial adhesion, such as the hydrophobicity of the implant or its surface tension and electrostatic forces [6]. The gradual formation of an EPS could lead to the inability of the immune cells to fight with bacteria, as well as to resistance to antibiotic treatment. Thus, a chronic inflammation could occur, resulting in an implant failure.

The limitations of the materials currently used for bone healing include the need for alternative materials of synthetic or natural origin that can overcome the above-mentioned issues, as well as the need to improve the process of bone tissue regeneration. Such materials must be suitable in terms of mechanical strength, biocompatibility, and physical or chemical similarity to bone tissue [7]. Furthermore, biomaterials for bone grafts should possess appropriate porosity and degradation rates in order for them to allow the natural bone formation and resorption processes to occur [8]. Biocompatible ceramics represent a group of materials that meet these requirements and have found a wide application in bone healing. For example, calcium silicates, and particularly wollastonite-based composites, have shown great mechanical and thermal properties, as well as excellent biocompatibility. The presence of Ca^2+^ and SiO_3_^2−^ ions improves the osseointegration and osteoconductivity of the material in in vivo models [9]. Such materials allow manipulation via different technological methods in order to achieve various designs for tissue engineering scaffolds. For instance, the group of Papynov et al. demonstrated a novel approach to the development of an osteoplastic porous wollastonite/hydroxyapatite scaffold, and monitored its integration in jaw-defect models in rabbits [10]. The researchers showed that the produced materials lacked in vitro cytotoxicity, while computed tomography scanning revealed the successful integration of the scaffold into the injured tissues of the animals. Another example of bioceramics is calcium phosphates. The most commonly used representatives of this group are β-tricalcium phosphate and hydroxyapatite. The main advantage of these materials is that their chemical structure closely resembles the mineral phase of natural bone tissue [11]. Hydroxyapatite (HA) exhibits a higher degree of crystallinity compared to native bone minerals, meaning that it has greater stability and lower degradability over time after insertion at the site of the injury [11]. On the other hand, β-tricalcium phosphate (β-TCP) is not solubilized under physiological conditions, but rather resorbed by mainly osteoclasts, and thus it could be gradually replaced by the newly forming bone in a process mimicking the dynamics of natural bone tissue homeostasis [12]. Another benefit of tissue engineering scaffolds based on calcium phosphates is that both their chemical and physical properties could be adjusted. For example, material porosity is a crucial feature for osteoinduction and osteoconduction. Wang et al. have investigated the effects of mean pore size on implant osseointegration in rabbits [13]. The study compared pore sizes ranging between 300 and 500 μm, and the results indicated that pores with a diameter of 400 μm and above proved to be optimal for bone cell proliferation. Furthermore, macroporosity also reduced the formation of cell aggregates. The literature on the topic also shows that pores with a diameter greater than 100 μm allow for the better diffusion of nutrients and waste removal between the inner side of a scaffold and the surrounding tissue [1]. Both micro- (<50 μm) and macroporosity in ceramic scaffolds have been found to play a major role in the mechanical stability of scaffolds [14]. Despite having major advantages for bone tissue engineering, calcium phosphate-based scaffolds could still be colonized by bacteria after implantation [15]. The material itself does not possess intrinsic bactericidal properties, thus various strategies for functionalization such as peptide immobilization, antibiotic loading or ion releasing need to be employed. For example, antibiotics have been proven as an effective tool for fighting various bacteria. Implants impregnated with antibiotics have shown good results against bacterial infections; however, there is no strong evidence on the effect of antibiotics for prolonged periods of time [16]. Furthermore, a major concern regarding the common use of antibiotics remains the potential for antibiotic resistance. Antimicrobial peptides (AMPs) are a potent alternative to antibiotics as they could harm both Gram-positive and Gram-negative bacteria through different mechanisms [16]. For implant functionalization, the AMPs could be attached to the surface via polymeric layers/hydrogels (i.e., allyl glycidil ether polymer brush) that serve to anchor the AMPs to the implant. Another mechanism for attachment could be covalent binding, which requires a particular AMP design in order for the binding to be successful and long-lasting. For instance, a defensin-based AMP has been attached to hydroxyapatite both covalently and electrostatically, and the antibacterial effects have been observed to preserve its efficacy for 3 weeks. Some limitations of the use of AMPs include the requirements regarding the initial design of the peptide, the need for the development of an anchorage layer over the implant, as well as the loss of activity, lower specificity, and sensitivity to the surrounding environment [16,17]. Nanoparticles of metal oxides have also been under investigation as part of another strategy to combat implant-associated bacterial infections. For example, zinc and copper oxide nanoparticles have been known to be effective against a broad range of bacteria and to exert their cytotoxicity via the direct uptake and generation of reactive oxygen species within the bacterial cells [18,19]. Nanoparticles can be deposited on the implant’s surface as a layer via, for example, sputtering or cathodic arc deposition [20]. After deposition, metallic ions undergo gradual release and interact with cells. Furthermore, nanoparticles could exert beneficial effects on the host cells as well. Concerning bone tissue engineering, zinc plays a role in bone mineralization as well as in the maintenance of cell membrane integrity [21].

A potent method that is used for controlling surface properties of materials without inducing any damages to their structural integrity is ultra-short laser structuring. The main advantage of this technique is the reduced formation of heat-affected zones that can potentially have a destructive influence on the material. During light–matter interaction, the thermal energy distribution is first transferred to the free electrons, and the time required for the energy to dissipate to the lattice spans picoseconds [22]. Thus, laser pulses lasting longer than this timeframe induce the strong melting of the material, inducing the redeposition of particles over the surface, resulting in damages to any arising adjacent structure [23]. In the case of ultra-short laser pulses, the duration of a single pulse can range between a few and a few hundred femtoseconds (fs), remaining below the temporal threshold for thermal energy dissipation. It has been postulated that the main mechanism of ablation in fs lasers is based on multiphoton ionization, during which the excited electrons enter a metastable quantum state and then become free electrons [24]. The electrons that participate in the multiphoton ionization are heated up significantly, and during energy transfer between them and ions of the lattice, occurring after the laser pulse, the ions’ temperature increases sharply. This leads to the vaporization of the material with a very short melting phase [24]. Ultra-short laser surface processing could create topographical patterns at the micro or nano level. For example, Chen et al. demonstrated the structuring of SiC using a femtosecond laser (λ = 800 nm; τ = 70 fs) [25]. Their study showed the simultaneous formation of both micro- and nanopatterns, with the latter representing laser-induced periodic surface structures (LIPSS). In their study, Ackerl et al. used a laser with 400 fs pulse width to process ceramic scaffolds, and observed that the processing did not lead to any major transitions in the phase of zirconia, thus confirming a key advantage of ultrashort laser processing—superficial modification without the alteration of the chemical structure [26]. The micro- or nanopatterning of surfaces by means of ultrashort laser ablation does not only re-form the local topography of a given material, but also leads to a shift in the roughness. Surface roughness is an important feature that has an influence on the properties of the material, such as wettability [27]. The research of Li et al. demonstrated how the use of an ultrashort laser with a central wavelength of 800 nm and pulse duration of 130 fs for the texturing of titanium plates can alter their surface roughness [28]. The results of this study showed that an incremental change in the laser fluence led to an increase in the Ra parameter (indicating the arithmetic mean roughness), thus acquiring highly hydrophilic surfaces. Again, ultrashort laser processing can be used to adjust a material’s properties by modifying it at a microlevel without affecting its structural integrity.

The present work proposes a laser-based methodology for the surface functionalization of ceramic scaffolds, aiming at a reduction in initial bacterial adhesion. To achieve this goal, an interdisciplinary approach to the development, processing and functionalization of the ceramic scaffolds has been chosen. Tricalcium phosphate-based scaffolds with controlled microporosity were surface-processed by ultra-short laser irradiation for the structuring of spatially defined micropatterns. Thus, the local surface roughness could be adjusted for improved cell adhesion and increased surface area. Then, the laser-modified surfaces were further functionalized with ZnO nanolayers by pulsed laser deposition in order to create a surface exhibiting potential antimicrobial activity without affecting the laser modified surface topography. The obtained modifications were tested against *S. aureus* for the investigation of early bacterial adhesion.

## 2. Materials and Methods

### 2.1. Production of Ceramic Scaffolds with Controlled Porosity Using a Sacrificial Phase 

The manufacturing of the scaffolds followed already established experimental protocols described by Yildiz et al. and Descamps et al. [29,30]. For this reason and also because all post-manufacturing modifications were exclusively restricted to the surfaces of the scaffolds, no experiments concerning the structure of the scaffold were conducted (i.e., porosity or density measurements). In brief, β-tricalcium phosphate powder was synthesized by aqueous precipitation using solutions of diammonium phosphate (NH_4_)_2_HPO_4_ (Carlo Erba, Val de Reuil, France) and calcium nitrate Ca(NO_3_)_2_**.**_4_H_2_O (Brenntag, France). The experimental conditions and the quantity of the used reagents were adjusted to obtain a final product containing 6 wt. % hydroxyapatite. The powder was calcinated at 850 °C for 3 h and then ground for the removal of possible agglomerates in a resin milling jar using yttria stabilized zirconia (YTZ) milling balls.

Aqueous slurries of the ground β-TCP were prepared with a 65 wt. % powder concentration. Commercial organic agent Darvan C (Vanderbilt, NY, USA) with 1.5 wt. % relative to powder quantity was used for the deflocculation process. Additionally, 4 wt. % organic binder (B1000 Duramax) for promoting the consolidation of the material during the following debinding process and 0.01 wt. % of Dynol surfactant (Evonik Resource Efficiency GmbH, Essen, Germany) to reduce surface tension were added to the initial mixture. Then, a slurry was obtained after 1 h of planetary milling with YTZ balls. The final product was poured over a polymethylmethacrylate (PMMA) scaffold in a plastic molder and left overnight to infiltrate the scaffold and to dry. 

The polymeric scaffold based on PMMA was created prior to the ceramic slurry production in the following manner, again based on previous research [29,30]: 1 g of PMMA beads (Diakon, Ineos Acrylics, Rotterdam, The Netherlands) with diameter 300–400 μm was inserted in a mold with a diameter of 16 mm. In order to induce the partial melting and controlled fusing of beads at their contact points, acetone was carefully poured on them. To stop the reaction, the polymeric frames were immersed in distilled water and then left to dry overnight. 

In order to obtain a final porous hydroxyapatite containing β-TCP scaffold (TCP-HA), first, the PMMA beads were thermally eliminated by heating up to 200 °C for 30 h at 1°/min, followed by dwelling at 400 °C for 5 h. Finally, the scaffolds were sintered at 1050 °C with a rate of 5°/min with dwelling at 1050 °C for 3 h. Thus, a cylindrical scaffold (h: 0.9 cm; w: 1.3 cm) with a dense outer surface and an inner porous core was prepared.

### 2.2. Development of Surface Microstructures via Ultrashort Laser Ablation

Surface processing of the scaffolds was achieved by ablation with a Ti:sapphire (Solstice Ace, MKS Spectra-Physics, Milpitas, CA, USA) femtosecond laser with a central wavelength λ = 800 nm and pulse duration τ = 70 fs. The repetition rate (ν) for all experiments was set at 1 kHz. All samples were arranged on a vertical XY translation stage (Thorlabs, NJ, USA), perpendicular to the direction of the laser beam. The velocity of the raster scans performed over the surface of the scaffolds was controlled by Kinesis^®^ Software v1.14.45 (Thorlabs, Newton, NJ, USA). The working laser parameters chosen for the experimental methodology were as follows: F = 24.4 J/cm^2^; v = 7.6 mm/s; d_x_ = 60 μm. Additionally, in order to observe whether an increase in laser fluence would lead to a phase transition on the surface of the material, the following ranges of working parameters were used: F = 20.3–50.9 J/cm^2^; v = 3.44–7.6 mm/s and d_x_ = 35–70 μm.

### 2.3. Deposition of ZnO Nanolayer over Ceramic Scaffolds

The surfaces of both laser-treated and untreated scaffolds were coated with a ZnO nanolayer by standard pulsed laser deposition (PLD). For this purpose, a home-made ZnO target was irradiated using a Q-switched Nd:YAG laser (Lotis TII, Minsk, Belarus) operating at 10 Hz with a wavelength (λ) of 355 nm and producing a pulse with a duration (τ) of 15 ns. The laser fluence applied was 1.5 J/cm^2^.

The deposition process was carried out at room temperature in an oxygen atmosphere at 0.01 mbar for 10 min. The distance between the target and the scaffolds was set at 4 cm. Due to the porous and uneven surface of the ceramic scaffolds, the thickness of the formed ZnO nanolayer was measured on a silicon substrate after performing PLD using the same parameters as for the scaffold functionalization. Ellipsometry measurements were performed using a Woollam M2000D ellipsometer in the 193–1000 nm wavelength range. The data acquisition and modeling software used was the CompleteEASE^®^ software v4.92. A two-layer model consisting of silicon substrate, with a 3.1 nm-thick silicon native oxide layer (as a first layer) and a ZnO layer represented by a Cauchy layer (as a second layer), was used to model the experimental ellipsometry data. In order to further chemically characterize the nanolayer formed on the ceramic scaffolds, X-ray photoelectron spectroscopy (XPS) was performed using an AXIS Supra electron spectrometer (Kratos Analytical Ltd., Manchester, UK), utilizing AlKα radiation with energy of 1486.6 eV. The values of the binding energy (BE) were calculated with an accuracy of ±0.1 eV using the C1s line (adventitious carbon) at 284.8 eV as a reference. In order to better determine the chemical state of Zn, the modified Auger parameter α^’^ was calculated via the following equation:

$$\alpha^{\prime }=ΚΕ\left(ΖnL_{3}M_{4,5}M_{4,5}\right)+BE(Zn2p_{3/2})$$

where *KE*(*ZnL*_3_*M*_4,5_*M*_4,5_) and *BE*(*Zn*2*p*_3/2_) represent the kinetic and binding energies, respectively, of the Auger transition, and the electron core level is indicated in the brackets. XPS was further used to calculate the concentration of the chemical elements (presented further as atomic %). This was performed by normalizing the areas of the photoelectron peaks (O1s, Ca2p, P2s and Zn2p_3/2_) to their relative sensitivity factors using the commercial data processing software ESCApe^TM^ v1.2.0.1325, Kratos Analytical Ltd. 

### 2.4. Characterization of Structural Changes after Ultrashort Laser Ablation

The effects of the laser machining on the phase and crystallinity of the material were evaluated via X-ray diffraction (XRD) and a micro-Raman analysis. The former analysis was executed via a Philips PW1050 X-ray diffractometer system (Philips, Amsterdam, The Netherlands) using a copper anode with a secondary monochromator of the diffraction beam. The range of measurement was 5–90° 2θ with a 0.05° 2θ step size. The acquired results were analyzed via QualX2 and Crystallography Open Database. During the XRD experiment, an automatic powder diffractometer system Empyrean (Malvern, Panalytical, Almelo, The Netherlands) equipped with an X-ray tube Cu anode and a PIXcel3D detector was used. Data were collected using a 0.03 2θ step size and 100 s exposition in 1D mode of the detector. Qualitative phase analysis was performed in the HighScore Plus software version 3.0e and ICSD (Inorganic Crystal Structure Database) provided by FIZ Karlsruhe GmbH, Leopoldshafen, Germany [31,32]. Quantitative ratios of the presented phases were obtained by Rietveld refinement [33]. The second analysis was carried out using a micro-Raman spectrometer (LabRAM HR Visible, Horiba, Kyoto, Japan) equipped with a He–Ne laser (633 nm) and a microscope (BX41, Olympus, Tokyo, Japan). The results were analyzed via Spectragryph v1.2.11 and Origin v9.5 softwares as all graphs have been baseline-corrected. In terms of morphological analysis, the scaffolds that were modified with varying parameters of laser fluence, scanning velocity and hatch distance (d_x_) were observed via scanning electron microscopy (“Lyra”, Tescan Orsay Holding, Brno-Kohoutovice, Czech Republic; TM4000 Hitachi High-Tech Europe). Prior to observation, the samples were coated with a thin layer of carbon. All images were obtained at 10 kV. An additional analysis by means of an optical profilometer (Zeta-20, Zeta Instruments, Gallatin Valley, MT, USA) was performed in order to evaluate the change in surface roughness after fs laser irradiation. The analysis involved the use of the Zeta Optics Module, which allowed a vertical Z scan, thus acquiring a 3D image in true colors. The applied field of view was 476 × 357 μm, with magnification of 20× used for all images. For the assessment of the surface roughness of the acquired 3D true-color images, Sa values were taken into consideration (Sa: arithmetic mean height; ISO 25178 [34]). The software used for image analysis was ProfilmOnline (www.profilmonline.com, accessed on 25 October 2023). 

### 2.5. Antimicrobial Activity of Functionalized and non-Functionalized Hydroxyapatite Containing TCP Scaffolds

The antibacterial activity of the ceramic scaffolds was tested against *Staphylococcus aureus* (ATCC 25923). Samples that were used for the in vitro experiments were the following: control scaffolds that were not subjected of either laser processing or pulsed laser Zn deposition (referred to as control); control scaffolds with ZnO that did not bear laser-induced patterns but were functionalized with ZnO (referred to as control ZnO); scaffolds with laser processing and ZnO deposition (referred to as laser ZnO); laser-processed scaffolds that did not undergo further functionalization with ZnO (referred to as laser no ZnO). The upper surfaces of the materials were exposed to a bacterial suspension (700 μL) of 10^6^ Colony-Forming Units (CFU)/mL, prepared in tryptic soy broth (TSB) (Liofilchem, Italy) for 3 h and 6 h at 37 °C. Antibacterial activity was evaluated for the sessile bacteria (attached to the material surface) and planktonic population (freely floating cells). Sessile bacteria were evaluated for cell morphology and colonies structure via scanning electron microscopy (SEM) and helium ion microscopy (HIM); additionally, the Resazurin assay was used to evaluate the metabolic activity. The planktonic bacteria were also assessed for metabolic activity, using the same assay.

#### 2.5.1. SEM and HIM Observation

Scaffolds were fixed for 30 min in 3% (*v*/*v*) glutaraldehyde (TAAB laboratories equipment Ltd., Aldermaston, UK), diluted in cacodylate buffer (0.14 M, pH 7.4, Thermo Scientific, Waltham, MA, USA) and dehydrated with increasing ethanol solutions. Scanning electron microscope (SEM) images were obtained using a Crossbeam 350 (Zeiss, Oberkochen, Germany) with an acceleration voltage of 5 kV, an aperture size of 15 µm and a work distance between 6 and 13 mm to prevent surface charging. Due to the sizes of the samples and to further minimize surface charging, the samples were coated with a 10 nm carbon layer to enhance electron flow. Before coating, energy-dispersive X-ray spectroscopy (EDX) measurements were taken with an Ultimax 170 detector (Oxford Instruments, Oxford, UK) to extract information about the sample composition and element distribution. To further assess the surface morphology of the samples and bacteria, an Orion Nanofab Helium Ion Microscope (Zeiss, Germany) was used to image regions of interest previously identified from the SEM images (beam energy: 25 keV, beam current: 0 to 3.15 pA). This allowed imaging with clear magnifications of up to 28,500× without major artifacts. The flood gun in line averaging mode was used to overcome sample charging.

#### 2.5.2. Metabolic Activity 

After each time-point, the metabolic activity of both the sessile and the planktonic population was evaluated via the Resazurin assay. For sessile bacteria, the colonized scaffolds were retrieved and washed with saline solution (0.9%, Biochem Chemopharma, Cosne-Cours-sur-Loire, France) and further incubated with 10% Resazurin diluted in TSB. Fluorescence was measured upon initial color change. For the assessment of planktonic bacteria, 90 μL of bacterial suspension from each well was mixed with 10 μL of Resazurin solution (0.1 mg/mL, Resazurin sodium salt, Sigma-Aldrich R7017, St. Louis, MO, USA), and incubated for 1 h at 37 °C. Fluorescence measurements were taken at 530 nm excitation/590 nm emission using a microplate reader (Synergy HT, Biotek, Winooski, VT, USA) with Gen5 1.09 Data Analysis Software.

### 2.6. Statistical Analysis

Results from the in vitro tests were analyzed by one-way ANOVA with Tukey’s post hoc test. Analyses yielding a *p* value ≤ 0.05 were considered to be statistically significant.

## 3. Results

### 3.1. Effects of fs Laser Treatment on TCP Scaffolds Containing 6 wt% of HA

**Figure 1:**
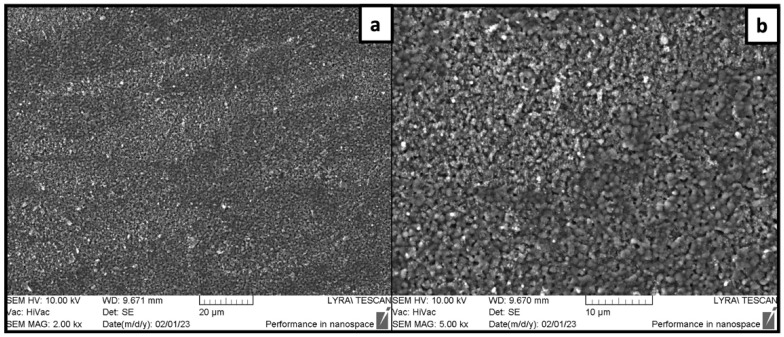
Dense outer surface of the TCP-HA scaffolds prior to laser ablation. (**a**) image at 2000× magnification; (**b**) image at 5000× magnification.

**Figure 2:**
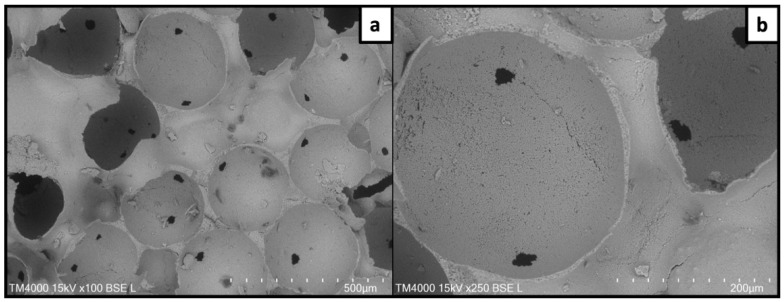
Internal porosity of the scaffolds as a result of PMMA beads’ thermal removal. (**a**) image at 100× magnification; (**b**) image at 250× magnification.

The impact of the fs laser treatment was first analyzed via scanning electron microscopy to assess the alterations in surface morphology. Figure 1 and Figure 2 are representative images of the two phases of the scaffold—the outer dense one and the inner porous phase, respectively. Figure 2 shows how the pores were formed after the thermal removal of the PMMA, retaining a spherical shape with clear interconnections between the adjacent pores. As mentioned above, the sizes of the polymeric beads used were 300–400 μm, while the average size of the pores in the final product after sintering was 270 μm.

**Figure 3:**
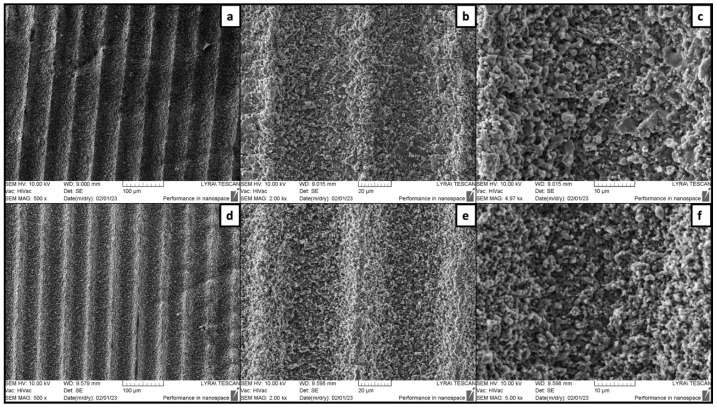
Femtosecond laser-patterned outer surfaces of TCP-HA. (**a**–**c**) F = 50.9 J/cm^2^; v = 5.16 mm/s; dx = 60 μm; (**d**–**f**) F = 30.6 J/cm^2^; v = 5.16 mm/s; dx = 60 μm.

**Figure 4:**
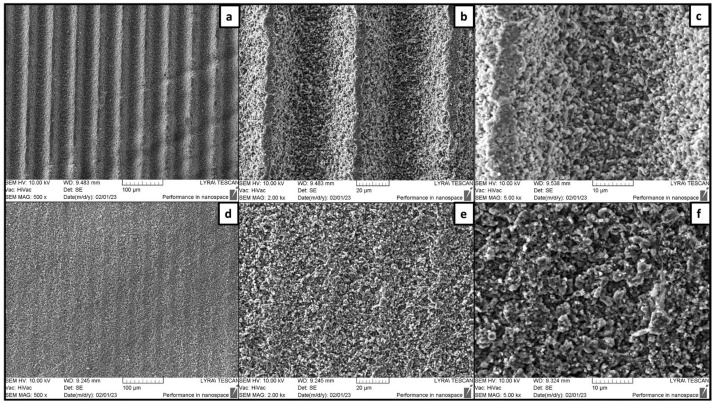
Femtosecond laser-patterned outer surfaces of TCP-HA. (**a**–**c**) F = 24.4 J/cm^2^; v = 7.6 mm/s; d_x_ = 60 μm; (**d**–**f**) F = 20.3 J/cm^2^; v = 3.44 mm/s; d_x_ = 35 μm.

Figure 3 and Figure 4 illustrate how the surface topography varied according to changes in applied laser fluence and hatch distance. Overall, a hatch distance kept at 60 μm and above allowed the formation of distinct parallel microchannels, regardless of the laser fluence (Figure 3). An increase of the hatch to 70 μm did not lead to any evident changes in the overall structure and the morphology at the bottom of the patterns (Figure 4a–c). However, narrow untreated smoother areas were formed on the ridges between two adjacent microchannels (Figure 4b,c). As shown in Figure 4d–f, when d_x_ was lowered to 35 μm, a more homogenous surface was obtained. The fs laser treatment led to a change in the surface porosity of the scaffolds as more micro cavities had formed, resulting from material etching and rearrangement. These results confirmed that by adjusting the hatch distance, it could be possible to obtain alternating rough and smooth surfaces simultaneously. Furthermore, no collateral heat-induced damage was observed in the zones surrounding the microchannels, thus preserving clear borders of the obtained modifications.

**Figure 5:**
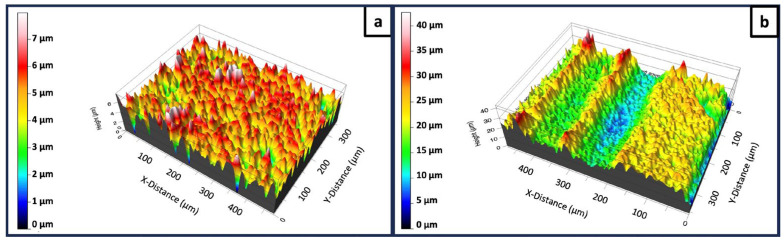
The 3D profiles of TCP-HA scaffolds. (**a**) An untreated surface; (**b**) an fs laser-treated area (F = 24.4 J/cm^2^; v = 7.6 mm/s; d_x_ = 60 μm).

Further insights on the effects of the fs laser irradiation on the surface of the scaffolds were obtained via an optical profilometer. Figure 5 shows the 3D profiles of an untreated and a laser-treated area, based on which surface roughness measurements were made. The two profiles, as expected, followed the morphology revealed by the SEM imaging shown above. The results indicate a clear difference, in that the Sa values of the area within the laser-induced microchannels (Sa = 1.85 μm) were substantially higher than the ones of the control areas (0.63 μm). This increase in the surface roughness could be attributed mainly to material ejection and rearrangement at the ablation zones. The presence of a rougher surface could mean a larger surface area, and it could potentially have an impact on cell adhesion.

### 3.2. Phase and Crystallinity Analysis of Ceramic Surfaces after fs Laser Processing 

**Figure 6:**
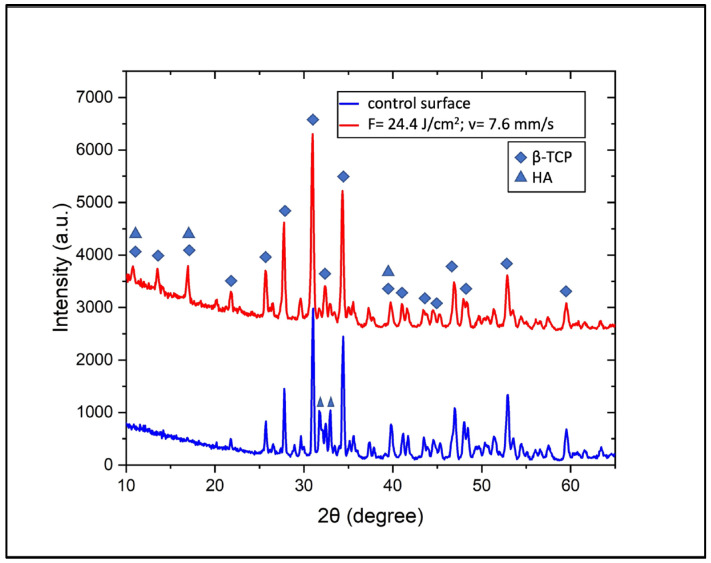
An XRD analysis indicating the presence of a β-phase of tricalcium phosphate before and after femtosecond laser treatment.

For the assessment of any changes in the phase transition or the crystallinity of the scaffolds at a surface level, XRD and micro-Raman analyses were carried out. XRD analyses were performed on scaffolds modified with the laser parameters chosen for further in vitro studies (F = 24.4 J/cm^2^; v = 7.6 mm/s). The yielded results did not reveal any alterations in the chemical structure of the ceramic material after the development of the surface modifications, as the β phase was preserved. The peaks characteristic of β-TCP and HA detected by XRD were analyzed via the aforementioned software, and were also confirmed by data seen in the literature [35]. The results are presented in Figure 6. There were evident differences between laser-processed and unprocessed surfaces, more specifically in the peaks between 30° and 35°. Overall, the intensity of the signal arising from the laser-treated surfaces was higher. The main characteristic peaks of β-TCP were preserved, as very slight broadening was observed. The surface treatment led to a rise in the three peaks in a range between 10° and 20°, with the peak at 13.5° being exclusively characteristic off β-TCP. Furthermore, a distinct difference was observed in the peak of β-TCP at 32.4°, which exhibited a sharp increase in intensity compared to the two adjacent peaks of HA at 31.7° and 32.9°. Additional details on differences between the two surfaces were obtained by comparing the quantitative ratios of the phases using the Rietveld refinement. The results show an increase in β-TCP from 79.2% in the control surface to 97.8% in the fs laser-treated surface. After laser ablation, the ratio of HA decreased sharply from 20.8% to 2.2%. The observed results could be explained via an increase in the crystallinity of β-TCP as a result of fs laser treatment. Femtosecond laser pulses deliver very high amounts of energy, leading to the localized heating of the material for a very limited period of time. This, combined with electronic excitation occurring during fs laser–matter interaction, could lead to potential crystallization. Based on these factors and the results presented in Figure 6, it could be stated that the fs laser processing improved the crystallinity of the β-TCP scaffold at the surface level.

**Figure 7:**
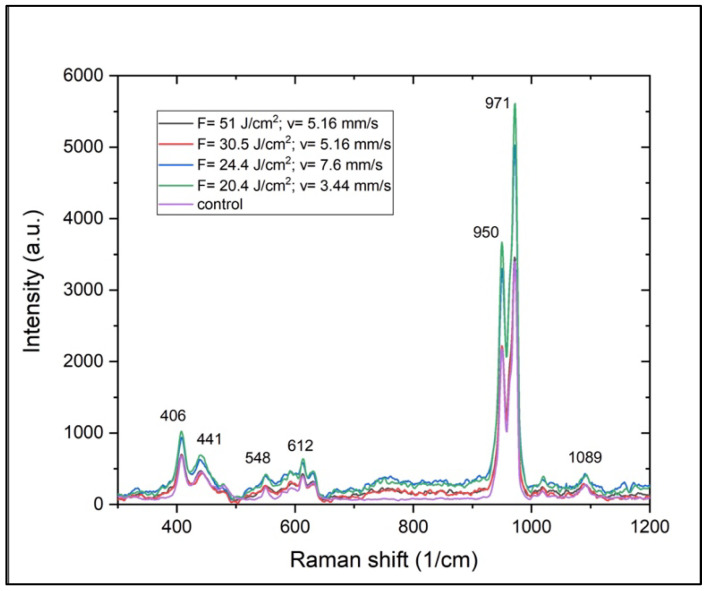
Raman spectra of TCP-HA scaffolds before and after femtosecond laser treatment with varying laser fluences applied at different scanning velocities.

The results yielded by Raman spectroscopy contribute to the observations that the fs laser radiation can be used to change the surface morphology of a ceramic material without altering its crystalline structure or inducing a phase transition. The Raman spectra (Figure 7) showed a difference only in the intensity of the detected characteristic peaks corresponding to treatments with varying laser fluence. Overall, the shapes and positions of the peaks were preserved, regardless of the surface treatment. Nandha Kumar et al. investigated structural and mechanical changes in tricalcium phosphates at varying heat treatments [36]. The authors reported Raman spectra of materials heated at 1400 °C and, similarly to the results reported in Figure 7, identified strong peaks at 406, 441, 548, 612, 949 and 969 cm^−1^ [36]. 

Both XRD and Raman analyses indicated that the ultrashort laser treatment of the ceramic scaffolds would allow surface structuring without significantly affecting the local crystallinity and phase of the material. 

### 3.3. Characterization of ZnO Nanolayer Produced by Pulsed Laser Deposition 

**Figure 8:**
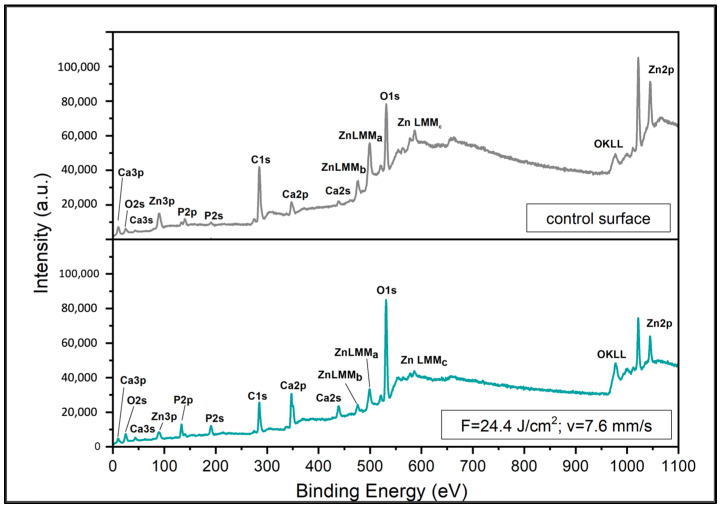
XPS survey spectra of ZnO-functionalized TCP-HA scaffolds. (**upper** panel) Control area; (**lower** panel) laser-treated area (F = 24.4 J/cm^2^; v = 7.6 mm/s).

**Figure 9:**
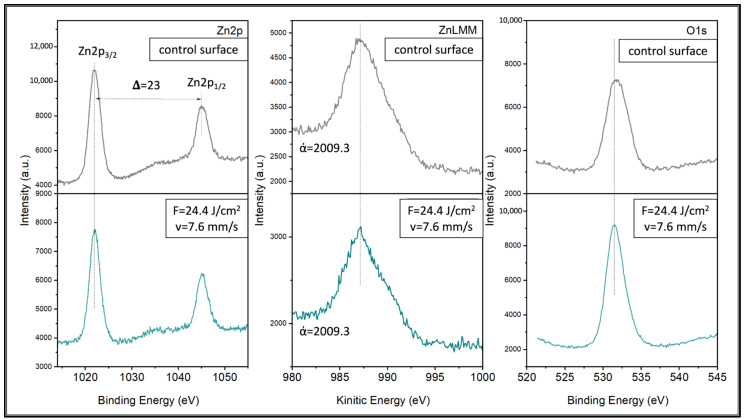
XPS spectra of ZnO nanolayer deposited on control and fs laser-treated surfaces of TCP-HA scaffolds.

The XPS survey spectra of both fs laser-treated and control areas confirm the presence of Ca, P, Zn, O and C, and reveal their distinctive peaks (Figure 8). A more detailed analysis of Zn identified the peaks of Zn2p_3/2_ and Zn2p_1/2_ at 1021.9 and 1044.97 eV, respectively, with a spin-orbit splitting of 23 eV (Figure 9). The spin-orbit splitting, the positions of the binding energies and the width of the Zn2p peaks clearly indicat that the Zn atoms were present in the Zn^2+^ oxidation state, as seen also in the work of Biesinger et al. [37]. This finding was further confirmed after the calculation of the Auger parameter, which was 2009.3 eV, corresponding to the same oxidation state of Zn (Figure 9) [38]. As mentioned earlier, the thickness of the ZnO nanolayer was measured on a silicon substrate after PLD with the same working conditions. The thickness was found to be 10 nm. XPS analysis confirmed the presence of a Zn^2+^ oxidation state and the same Auger parameter, while a survey spectrum did not detect another element apart from Zn and O.

In addition to the results presented in Figure 8 and Figure 9, the concentrations of the present elements were also determined (Table 1). Clear differences in the atomic % of Zn, P and Ca could be noticed. The concentration of Zn was nearly 3× higher in a control area when compared to the laser-structured one. This could be explained by virtue of the control area being flatter and smoother than the laser-treated one; thus, a stronger signal from the deposited Zn could be detected. This could be attributed to the morphological profiles of the two areas as the ZnO deposited on a surface without micropatterns and with submicron roughness could yield a stronger signal (Figure 5). As expected, the concentrations of Ca and P were higher in the micropatterned samples where a larger surface area was present. Overall, based on the results yielded by XPS, it could be concluded that the PLD of ZnO on the ceramic laser-treated scaffolds was successful.

### 3.4. Antibacterial Effects of Functionalized and Non-Functionalized Ceramic Scaffolds on S. aureus

TCP-HA scaffolds, unprocessed and laser-processed, and both with and without ZnO deposition, were analyzed for their antibacterial activity against sessile (attached) and planktonic (freely floating) *S. aureus* at 3 h and 6 h of incubation in the bacterial broth.

#### 3.4.1. Sessile Bacterial Population 

**Figure 10:**
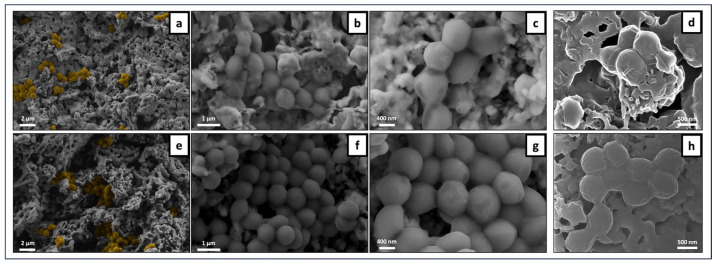
Representative SEM and HIM images of *S. aureus* adhered on unprocessed TCP-HA scaffolds at the 3 h time-point. Control samples: SEM (**a**–**c**) and HIM (**d**). Control ZnO sample: SEM (**e**–**g**) and HIM (**h**). (**a**,**e**) Bacterial cell coloring for a better visualization.

**Figure 11:**
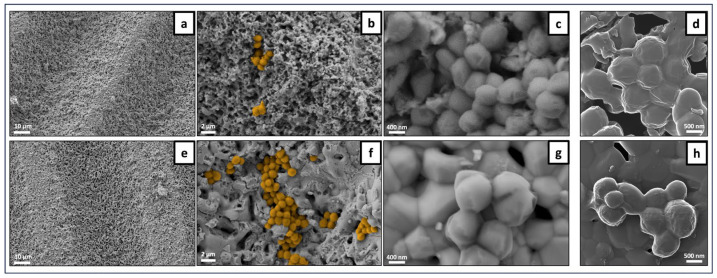
Representative SEM and HIM images of *S. aureus* adhered on laser-processed TCP-HA scaffolds at the 3 h time-point. Laser ZnO samples: SEM (**a**–**c**) and HIM (**d**). Laser no ZnO samples: SEM (**e**–**g**) and HIM (**h**). (**b**,**f**) Bacterial cell coloring for a better visualization.

**Figure 12:**
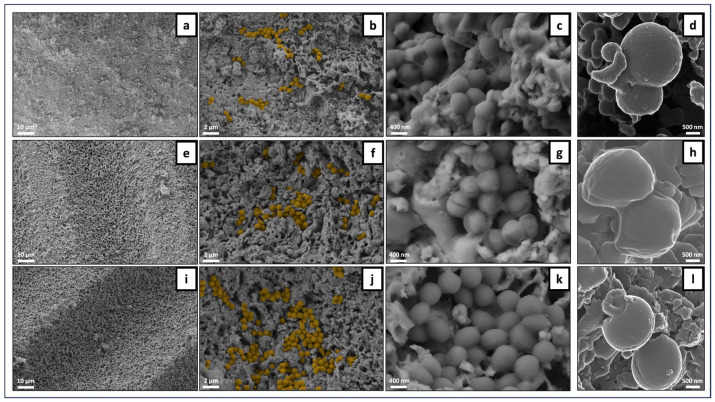
Representative SEM and HIM images of *S. aureus* adhered on TCP-HA scaffolds at the 6 h time-point. Unprocessed control: SEM (**a**–**c**) and HIM (**d**). Laser ZnO sample: SEM (**e**–**g**) and HIM (**h**). Laser no ZnO sample: SEM (**i**–**k**) and HIM (**l**). (**b**,**f**,**j**) Bacterial cell coloring for visualization.

The samples were incubated with *S. aureus* and analyzed for the adhered bacteria by SEM and HIM (Figure 10, Figure 11 and Figure 12). Overall, the SEM observation of the samples revealed a non-homogeneous clustering of small bacterial colonies at different sites across surfaces, regardless of laser processing, ZnO functionalization and incubation time. This is exemplified in representative images of the samples incubated for 3 h (Figure 10 for unprocessed samples without (a–d) and with (e–h) ZnO deposition, and Figure 11 for laser-processed samples with (a–d) and without (e–h) ZnO deposition). Images illustrating the sessile population at 6 h of culture time are shown in Figure 12 for control and laser-modified samples. As regards laser modifications, bacteria were growing mostly across the bottom of the microchannels and less on the vertical walls. In some samples it could be seen how bacteria preferentially grew on the more flat parts of the ridges between the microchannels (Figure 11e–g). Bacteria coloring (for better visualization) is suggestive of lower cell colonization on the ZnO-containing samples (Figure 11b,f and Figure 12f,j). HIM was used to assess more closely the morphologies of individual bacteria within the colonies. Overall, the images reveal cells with a varying surface roughness and embedded in an extracellular material within the small clustered colonies. However, it is worth highlighting the different behaviors regarding the cell morphology and the colonies’ structure between non- and ZnO-functionalized surfaces, both in unprocessed and laser-processed samples. Scaffolds without ZnO deposition exhibited colonies with relatively individualized cells, kept together by a thin layer of extracellular material, as shown in Figure 10d (control), Figure 11h and Figure 12h,l (laser-processed samples). Comparatively, in the ZnO-functionalized materials, i.e., control (Figure 10h) and laser-modified samples (Figure 11d and Figure 12h), the cells exhibited a rougher morphology kept together by a greater amount of involving material. These differences are particularly evident in Figure 12 (materials incubated for 6 h). Cells attached on the non-functionalized samples appeared round and smooth with defined cell limits (Figure 12d,l), whereas those present on the ZnO-functionalized samples displayed an irregular rounded and bumpy appearance, suggesting the leakage of cytoplasmic contents seen as a thick surrounding material (Figure 12h).

**Figure 13:**
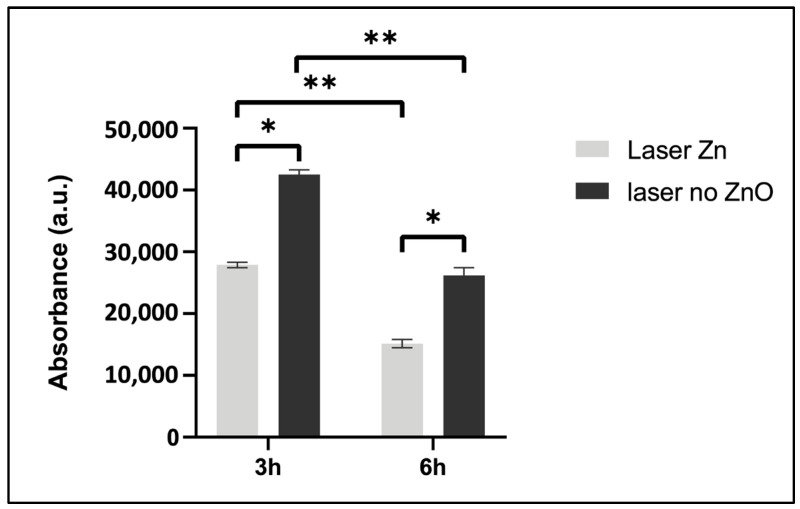
Metabolic activity of *S. aureus* sessile population 3 and 6 h after cultivation on laser-treated TCP-HA scaffolds with or without ZnO deposition. * Statistical significance (*p* ≤ 0.05) compared to ZnO samples. ** Statistical significance between 3 h and 6 h time points.

To further clarify the antibacterial effect of ZnO-functionalized materials suggested under SEM imaging, the sessile population was analyzed for its metabolic activity. Figure 13 summarizes the results observed in the laser-processed samples with and without ZnO deposition. Clearly, metabolic activity was significantly reduced on the laser ZnO samples at 3 h and 6 h of incubation time. Also, the antibacterial effect was greater with 6 h of culturing (on both surfaces), similar to that suggested in the SEM and HIM images. 

#### 3.4.2. Planktonic Bacterial Population

**Figure 14:**
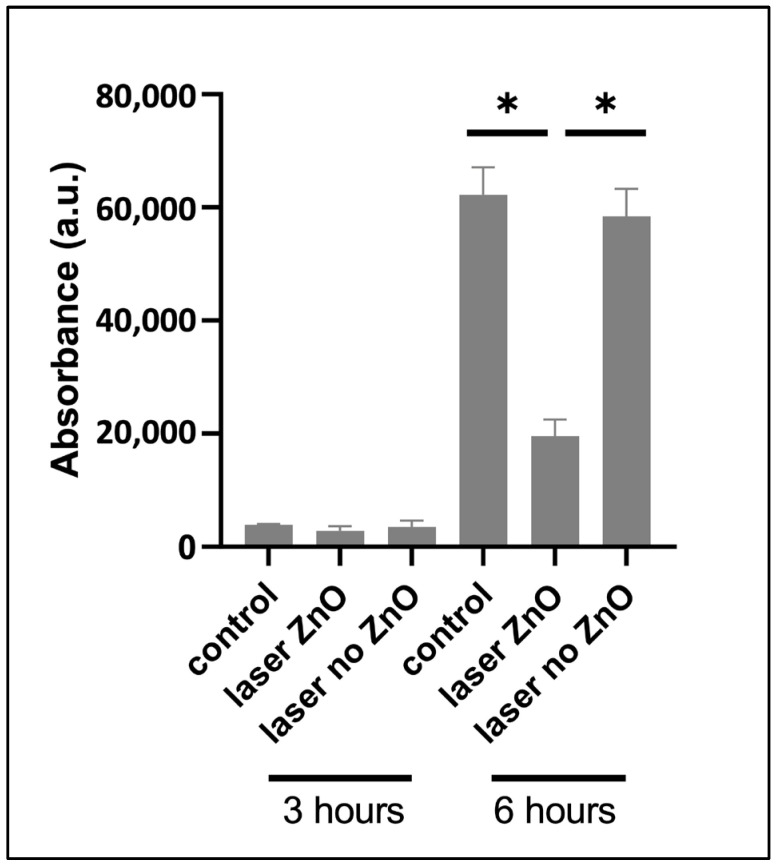
Metabolic activity of planktonic *S. aureus* (Resazurin assay) after 3 and 6 h of culture on TCP-HA scaffolds. * Statistical significance (*p* ≤ 0.05) compared to laser ZnO group.

The metabolic activity of planktonic bacteria (freely floating around the material samples) was evaluated at 3 and 6 h of incubation (Figure 14). Under control conditions, *S. aureus* presented a typical growth curve throughout the culture period, i.e., a lag phase during the first hours (3 h time point), followed by an exponential growth at longer incubation times (6 h time point). The tested materials presented similar behaviors during the lag phase, which was expected due to the low metabolic activity. Nevertheless, with exponential cell growth, characterized by a high and efficient metabolism, the laser-processed ZnO samples significantly reduced the metabolic activity of the planktonic population. This was not observed on the laser samples not bearing ZnO deposition. As such, the measured optical density values had risen 16× for control and laser no ZnO samples, while the respective values for the laser ZnO materials had increased 6×.

## 4. Discussion

The aim of this work was to combine two laser-based techniques, namely, ultrashort laser surface processing and pulsed laser deposition of ZnO, for the development of antibacterial interface on ceramic scaffolds for bone tissue regeneration. The employment of ultrashort laser texturing allows highly precise and localized surface patterning, as well as the control of surface roughness. Pulsed laser deposition is a method widely used for growing thin layers at a nanoscale that can exert particular effects as coatings without compromising the surface topology of the material. 

The scaffolds used in this work based on β-TCP containing 6 wt. % hydroxyapatite were designed, and they yielded a specific microarchitecture with controlled porosity. The purpose of the sacrificial PMMA scaffold was to yield an inner porous core with controlled porosity that would allow appropriate interconnectivity. Additionally, this inner core remained surrounded by a dense phase of the material formed during the drying process (see Section 2.1) and then stabilized after sintering. The chosen design of the ceramic scaffold could mimic the architecture of native bone tissue with its cancellous and compact parts, which, in combination with the beneficial effects of the calcium phosphates on bone regeneration, could effectively improve the tissue healing process. Panseri et al., whose scaffold design was followed in this paper, compared scaffolds with varying microarchitectures, including spongy, cortical and dual core–shells (composed of a more porous core surrounded by a lamellar freeze-casted layer), based on either hydroxyapatite or β-TCP [39]. The results demonstrate that all three designs induced osteogenic differentiation after the relative quantification of respective cell markers, with the main difference being in the type of calcium phosphate used. In addition to the structural design of the scaffolds described in the present paper, their dense outer part was further modified at a microlevel by ultrashort laser irradiation. By material etching and partial rearrangement, the surface processing induced the formation of parallel microchannels, which increased the surface roughness. This aspect of the surface topography has a great importance for cell–material interaction. Cell adhesion is the first phase during implant-mediated tissue regeneration, and serves as the basis for consecutive cell proliferation and differentiation [40]. By mechanical sensing, the cells can receive cues from the surrounding environment, which can then affect cell behavior and gene expression [41]. Thus, the cell activity can be modulated by controlling the surface topography. Regarding bone implants, it has been found that roughness on a micro or nano scale in certain ranges has a beneficial effect on cells in terms of adhesion and expression of bone markers. The paper of Stoilov et al. states that an optimal range of surface microroughness (Sa) for the improved osseointegration of an orthopedic implant is between 1.2 and 2 μm [42]. Osman et al. monitored the response of human osteoblasts to titanium with varying surface roughnesses [43]. Their results indicate that surfaces with Sa of 1.2–1.4 μm stimulated cell proliferation to a higher degree when compared to smooth (Sa: 0.08–0.1 μm) or rough surfaces (Sa: 3.3–3.7 μm). The data described in the previous chapters of the present paper reveal that the Sa value corresponding to the untreated surfaces (0.63 μm) was more than two times lower compared to the one measured at the laser-ablated zones (1.83 μm). Based on evidence in the literature, it is considered that the surface roughness within the laser-induced microchannels should improve the adhesion and proliferation of osteoblastic cells in future studies. Apart from the topographical alterations that the fs laser ablation can induce, it has also been found that it can shift the phase of tricalcium phosphate [44]. In order to monitor changes in the chemical structure of the material, Raman spectroscopy and XRD were performed. The results of these analyses confirm that the described methodology allowed surface structuring without any structural change in the elements.

As was described in the first section of this article, calcium phosphates have been established as a preferred material for the healing of bone defects. Despite their multiple benefits, due to their lack of intrinsic antimicrobial properties, the possibility for the development of bacterial infection after implantation still remains present. Additionally, it has been suggested that surface porosity could also have a stimulating effect on bacterial attachment due to the larger surface area [45]. Furthermore, dimensions of surface patterns and overall morphological features that are close to the sizes of bacterial cells could provide better attachment sites and shelter the cells from any mechanical stress, such as fluid flow [46]. In their article concerning bacterial attachment to ceramic implants, Kinnari et al. showed that *S. aureus* and *S. epidermidis* readily adhered to the surface of biphasic calcium phosphate at a physiological pH of 7.4 after 90 min of incubation; however, a drop in pH to 6.8 reduced the number of attached bacteria [47]. These results confirm that the ceramic material alone does not have any inhibiting effects on the bacteria; thus, additional factors would be required. In our study, the formed morphological patterns provided a surface that could improve bone cell adhesion and proliferation. PLD was chosen as a strategy for the nanocoating of ZnO over both laser-structured and unmodified surfaces in order to impart the surfaces with antibacterial properties. Via the nanosecond laser ablation of a ZnO target, a layer of ZnO with an approximate thickness of 10 nm was deposited on top of the TCP-HA scaffolds. The antibacterial activities of TCP-HA scaffolds were analyzed for sessile and planktonic *S. aureus* following incubation for 3 and 6 h.

The SEM observation of the adhered population on the material’s surface (Figure 10, Figure 11 and Figure 12) revealed smaller clusters of bacteria spread across different areas of the samples’ surfaces in a non-homogeneous manner. HIM imaging allowed a better visualization of the cell morphology within the colonies. On the ZnO-functionalized surfaces, cells displayed a rougher and bumpier surface, with a higher amount of surrounding material, compared to that seen on the materials not bearing ZnO deposition. This behavior could suggest a stress environment probably involving leakage of cellular contents hindering cell proliferation. In line with these observations, Mendes et al., in a work investigating the effects of ZnO nanoparticles on bacteria, suggested that the presence of ZnO readily induced membrane disruption in several bacterial species, including *S. aureus*, after 15 min of incubation at a pre-determined inhibitory concentration [48]. In another research, covering the same problem, Wahab et al. examined the antimicrobial activity of ZnO nanostructures with a distinct geometry. By using Bio-TEM (Bio-Transmission Electron Microscopy), the researchers visualized the adherence of ZnO nanostructures to the outer surfaces of *S. aurues* and their subsequent internalization by the cells [49]. Furthermore, the authors reported cell damage that had potentially been inflicted by leakage of cellular contents.

Further, the evaluation of the metabolic activity of the sessile population on TCP-HA samples showed a significant inhibitory effect in the laser-processed and ZnO-functionalized surfaces. A similar trend was verified regarding the planktonic population. A significant decrease in the bacterial metabolic activity was observed on the ZnO-containing samples (Figure 13 and Figure 14). These results may indicate that the presence of the ZnO nanolayer exerted an inhibitory effect on both sessile and planktonic bacterial populations. ZnO has been shown to affect bacteria through a number of mechanisms. Two of these involve: (i) the production of free Zn^2+^ ions from the Zn nanoparticles and (ii) the attachment of ZnO nanoparticles to the external part of the bacteria via electrostatic forces [50]. As an element, zinc has been found to be an important factor in multiple vital intracellular processes occurring in all organisms; however, an increase in its concentration could have a detrimental impact for cells [51]. Particularly in bacterial cells, it has been reported that rising levels of free zinc can result in protein deactivation, disruption in glycolysis, polysaccharide synthesis, acid tolerance and proton translocation [52]. It has been shown that ZnO nanoparticles preserve a positive charge in a pH range of 6.5–10, thus interacting strongly with the negatively charged bacterial surface, leading to cell destabilization and increased permeability [50]. Furthermore, Fulindi et al. stated that Zn^2+^ ions could protract the lag phase of bacterial growth [53]. This was also observed in the present study (Figure 11). Bacterial cells grown on ZnO-functionalized surfaces exhibited evidently lower metabolic activity at the sixth hour compared to the scaffolds bearing only laser modifications. Such evidence on metabolism impairment by ZnO could provide an explanation for our results regarding both sessile and planktonic bacteria.

Overall, this study suggests that both surface morphology and the presence of ZnO could contribute to the inhibitory effects on bacterial behavior. The results presented above demonstrate that additional functionalization via the deposition of ZnO would be an appropriate strategy for the realization of novel orthopedic implants, thus improving bone regeneration while exerting antimicrobial effects.

## 5. Conclusions

This article presents a laser-based methodology that aims at developing antibacterial interfaces on ceramic scaffolds for bone healing. The work focused on investigations of the initial adhesion of *S. aureus* to ceramic surfaces functionalized either by femtosecond laser-induced modifications or by ZnO nanolayers, or by both. The obtained results show that the combination of laser-induced surface patterns and ZnO led to a distinct reduction in the metabolic activity of *S. aureus*. By implementing further optimizations and improvements, we believe that the proposed experimental procedure could contribute to the resolution of the current issues in bone tissue engineering related to bacterial infections.

## Figures and Tables

**Table 1:** Concentration of elements identified in ZnO-functionalized TCP-HA scaffolds.

Surface Type	O (at.%)	Ca (at.%)	P (at.%)	Zn (at.%)
Control	65.4	6.7	6.5	21.4
Fs laser-treated (F = 24.4 J/cm^2^; v = 7.6 mm/s)	64.7	11.9	15.2	8.2

## Data Availability

Data are contained within the article.
